# Wnt3a protein overexpression predicts worse overall survival in laryngeal squamous cell carcinoma

**DOI:** 10.7150/jca.35009

**Published:** 2019-08-08

**Authors:** Diekuo Zhang, Guo Li, Xiyu Chen, Qiancheng Jing, Chao Liu, Shanhong Lu, Donghai Huang, Yunyun Wang, Pingqing Tan, Jie Chen, Xin Zhang, Yuanzheng Qiu, Yong Liu

**Affiliations:** 1 Department of Otolaryngology, Head and Neck Surgery, Xiangya Hospital, Central South University, 87 Xiangya Road, Changsha, Hunan 410008, People's Republic of China.; 2 Otolaryngology Major Disease Research, Key Laboratory of Hunan Province, 87 Xiangya Road, Changsha, Hunan 410008, People's Republic of China.; 3 Department of Head and Neck Surgery, Hunan Cancer Hospital, The Affiliated Tumor Hospital of Xiangya Medical School, Central South University, 283 Tongzipo Road, Changsha, Hunan 410013, People's Republic of China.; 4 Department of Otolaryngology, Head and Neck Surgery, Changsha Central Hospital, 161 Shaoshan Road, Changsha, Hunan 410004, People's Republic of China.

**Keywords:** Laryngeal squamous cell carcinoma, Wnt3a, Metastasis, prognosis

## Abstract

As a classical ligand in the canonical Wnt/β-catenin signaling pathway, the role of Wnt3a in laryngeal squamous cell carcinoma (LSCC) remains unclear. Therefore, the expression pattern of the Wnt3a protein in 222 primary LSCC, and 19 corresponding adjacent non-carcinoma specimens, was detected by immunohistochemistry and further correlated with clinicopathological parameters. The results showed that LSCC tissue expressed higher levels of the Wnt3a protein when compared to the corresponding adjacent non-cancerous tissues. High expression of Wnt3a was closely related to histological grade (P = 0.031), clinical stage (I+II / III+IV; P = 0.004), and lymph node metastasis (P = 0.03). Kaplan-Meier analysis evidenced that a worse overall survival (OS) was correlated to the group with high Wnt3a expression (P = 0.003). When stratified survival analyses were performed, patients with lymph node metastasis/advanced clinical stages and high Wnt3a expression had worse OS rates than patients with other features (P < 0.001). Finally, multivariate analysis showed that Wnt3a expression was an independent prognosis factor for LSCC patients. The current findings suggest that Wnt3a is tightly related to the LSCC progression and could serve as a valuable clinic biomarker for LSCC patients.

## Introduction

Laryngeal squamous cell carcinoma (LSCC) is the second most common head and neck type of cancer globally, being a major cause of the death from cancer-related diseases 1, 2. Recent decades witnessed that patients with LSCC have benefited from early diagnosis and multimodal interventions, including routine surgery, chemotherapy, radiotherapy, and biological therapy. However, clinical outcomes for patients with LSCC remain discouraging, especially in advanced cases 3-5. Cervical lymph node metastasis has been reported as a decisive factor contributing to the dismal prognosis in post-treatment patients 3, 4. Nevertheless, the molecular mechanism of cancer initiation and progression remains unclear. Therefore, clarifying sensitive and specific molecular markers that can predict clinical outcomes of patients with biologically aggressive neoplasms is of great significance for developing more effective strategies for the prevention and treatment of patients with LSCC.

The Wnt signaling pathway has an important role in embryonic development, tissue homeostasis, tissue damage repair, cell proliferation, differentiation, and apoptosis 6-8. Dysregulation of this signaling pathway is associated with a series of human diseases, including various human malignancies 6. In the canonical Wnt/β-catenin signaling pathway, diverse Wnt ligands bind to Frizzled receptors and low-density lipoprotein receptor-related protein (LRP-) 5 or 6, resulting in the accumulation of β-catenin in the cytoplasm; consequently, the stabilized β-catenin enters the nucleus, leading to transcriptional activation, promoting malignant behaviors, such as cancer stem cell (CSC) self-renewal, local or distant metastasis, chemo- / radio- resistance, and immune escape 6-10.

Wnt3a is a classical member of the Wnt family, which comprises 19 homologous ligands 6. The aberrant increase in Wnt3a expression level has been previously reported in several human malignancies and has been correlated with tumor progression, recurrence, and metastasis, predicting worse clinical outcomes 11. However, few studies deal with the expression pattern and prognostic significance of the Wnt3a protein in LSCC. To provide a deeper insight into the roles of Wnt3a in LSCC, 222 LSCC tissue samples were studied to detect Wnt3a expression level and their relationship to clinicopathological parameters.

## Materials and methods

### Patient information and tissue preparation

In brief, archival paraffin-embedded cancer tissue and adjacent non-cancerous epithelial tissue samples from 222 patients with LSCC were analyzed in this retrospective study, for which tissue specimens and related information were available and collected. The samples were collected from January 2000 to October 2010. Patients with LSCC enrolled in our study had to follow these inclusion criteria: primary laryngeal squamous cell carcinoma without other malignancies and no history of previous radiotherapy or chemotherapy. Recurrence and metastasis were established based on physical examination, imaging results, and pathological investigations. Pathological (TNM) stage was determined according to the 7th American Joint Committee on Cancer (AJCC) staging system. The main clinical and pathological variables of the patients such as sex, age, primary tumor site, histological grade, T classification, clinical stage, and lymph node metastasis are described in Table 1. Informed consent was collected from all patients with LSCC before surgery, and all experiments were approved by the Research Ethics Committee of Central South University, Changsha, China.

### Immunohistochemistry

Immunohistochemistry was performed as reported previously 12-14. In brief, antigen retrieval was carried out in 10 mM citrate buffer (pH 6.0) in a microwave oven for 15 min. The activity of endogenous peroxidase was exhausted with 3% hydrogen peroxide for 10 min at room temperature. Anti-Wnt3a polyclonal antibody (Cat. CSB-EL026136HU, Cusabio Technology, Houston, TX, USA) was applied overnight at 4 °C at an optimal working dilution of 1:600. After sufficient phosphate-buffered saline rinses, sections were immunostained with horseradish peroxidase-labelled goat anti-rabbit polymers. Finally, positive staining of Wnt3a protein was visualized with 3′, 3′-diaminobenzidine, and the cell nucleus was counterstained with Mayer's hematoxylin. For negative controls, we used immunoglobulin G in the place of the anti-Wnt3a antibody under the same experimental conditions and co-incubated the anti-Wnt3a antibody with recombinant Wnt3a polypeptide to confirm the specificity of the anti-Wnt3a antibody applied in our experiment.

### Evaluation of Wnt3a staining

All immunohistochemical slides were blindly assessed and scored by two independent investigators. If a disagreement occurred, the slides were re-examined to obtain a final consensus. A Wnt3a staining index (SI), ranging from 0 to 7, was finally determined as the sum of the values obtained for the staining intensity and the proportion of immunopositive cancer cells for each sample. Staining intensity was classified as 0 to 3, in which 0 point meant no intensity, 1 point meant weak intensity, 2 points meant moderate intensity and 3 points meant strong intensity. In addition, the proportion of immunopositive cancer cells was ranked into four groups: <10% = 1, 10-34% = 2, 35-70% = 3, and >70% = 4. The expression level of Wnt3a was categorized as low expression (SI = 0-2), middle expression (SI = 3-4), and high expression (SI = 5-7) based on the final SI values 15.

### Follow-up

In this patient cohort, metastasis was determined by imaging results and clinical and pathologic examination. Two hundred and seven patients with LSCC (93.2%) had a full-range post-operational follow-up, and intact clinical information was obtained. Only 15 patients failed their follow-ups because their addresses or telephone numbers were changed. Overall survival (OS) was calculated from the day of surgery to the date of death or tumor relapse. Death of patients with LSCC resulting from other causes was defined as censored cases.

### Statistical analysis

All statistical analyses were performed using the software SPSS, v.17.0 (SPSS Inc., Chicago, IL, USA). The χ² test was used to analyze the associations between various clinicopathological parameters and Wnt3a protein expression. Survival analysis was assessed by the Kaplan-Meier method and survival curves were determined by the log-rank test. Univariate and Multivariate analyses for independent prognostic indicators were performed by the Cox proportional hazards model. P < 0.05 was considered statistically significant.

## Results

### Wnt3a is overexpressed in LSCC specimens

**Figure 1:**
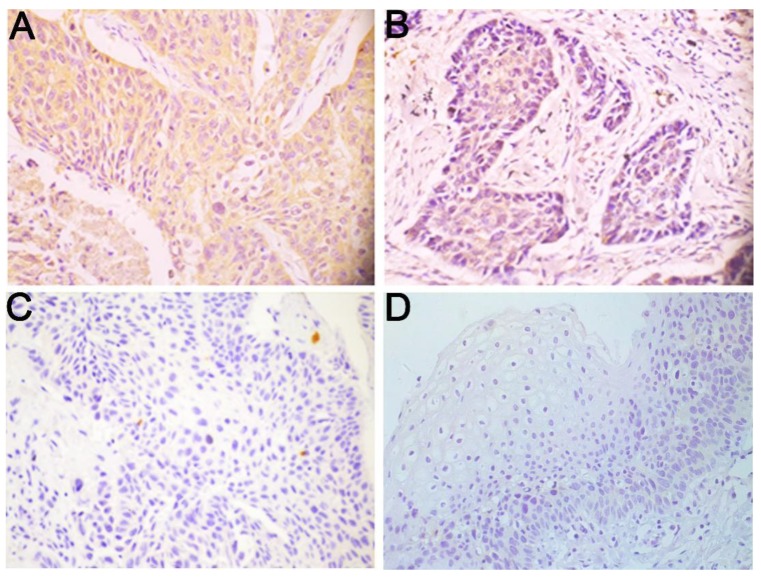
Wnt3a is overexpressed in LSCC. Representative immunohistochemical staining for Wnt3a protein in LSCC tissues and corresponding adjacent non-cancerous epithelial tissues. **(A)** High expression of Wnt3a protein in primary LSCC specimens. **(B)** Middle expression of Wnt3a protein in primary LSCC specimens. **(C)** Low expression of Wnt3a protein in **(C)** primary LSCC specimens and **(D)** negative expression of Wnt3a in adjacent non-cancerous epithelial tissues. Original magnification: 400×.

To elucidate the expression pattern of Wnt3a protein in LSCC, 222 paraffin-embedded, archival primary LSCC tumor specimens and 19 corresponding adjacent non-cancerous specimens were evaluated using immunohistochemistry. Wnt3a was predominantly distributed in the cytoplasm of LSCC cells (Fig. 1). Among these tumor samples, 52 (23.4%) patients displayed high Wnt3a expression, 95 (42.8%) patients with middle Wnt3a expression and 75 (33.8%) patients with low Wnt3a expression (Fig. 1A-C; Table 1). Wnt3a protein was dimly expressed in all the corresponding adjacent non-carcinoma samples (Fig.1D). These results indicate that Wnt3a is overexpressed in LSCC.

### Associations between the expression of Wnt3a protein and clinicopathological parameters

We further investigated the associations between Wnt3a expression and several clinicopathological parameters. As summarized in Table 1, Wnt3a overexpression was significantly associated with histological grade (P = 0.031), clinical stage (P = 0.004), and cervical lymph node metastasis (P = 0.030). However, no significant correlation was observed between the expression of Wnt3a protein and parameters such as age (P = 0.746), gender (P = 0.095), tumor site (P = 0.776), and tumor T classification (P = 0.356). These results suggest that Wnt3a expression is closely related to the progression of LSCC.

### Wnt3a expression is correlated with poor clinical outcome in LSCC patients

**Figure 2:**
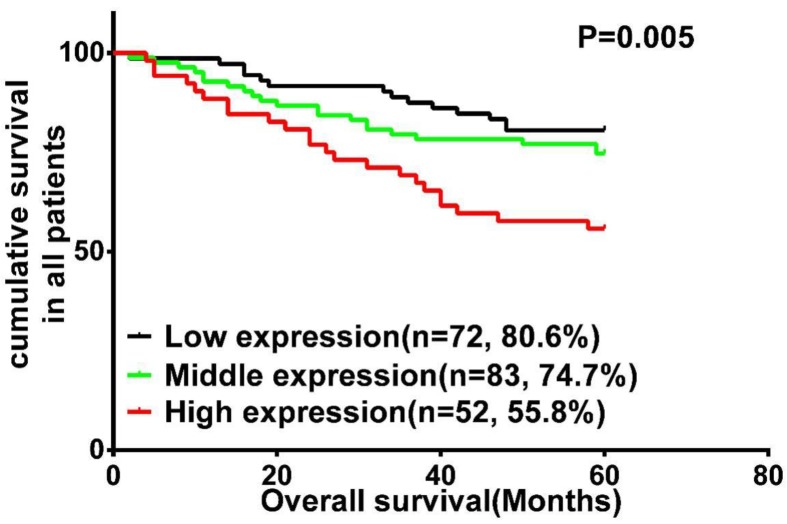
Kaplan-Meier analysis and log-rank test for overall survival in LSCC patients with differential expression of Wnt3a.

A survival analysis was performed using data from 207 patients with LSCC, for whom complete clinical outcome information was available. We categorized the whole cohort of patients with LSCC into three groups: high (n = 52), middle (n = 83) and low (n = 72) Wnt3a expression. Kaplan-Meier survival analysis demonstrated that LSCC patients with high Wnt3a expression had an increased risk of overall mortality (P = 0.005; Fig. 2). Log-rank test further confirmed that the difference in 5-year OS rates among these three groups was statistically significant. Univariate Cox regression analyses showed that primary tumor site, clinical stage, T classification, lymph node metastasis, and Wnt3a protein were significantly associated with OS (All P < 0.05). Clinical stage and Wnt3a protein expression were finally determined to be independent factors with prognostic value for OS (P = 0.037 and P = 0.024, respectively) in patients with LSCC via multivariate Cox analyses (Table 2).

### Stratified survival analysis based on Wnt3a expression

**Figure 3:**
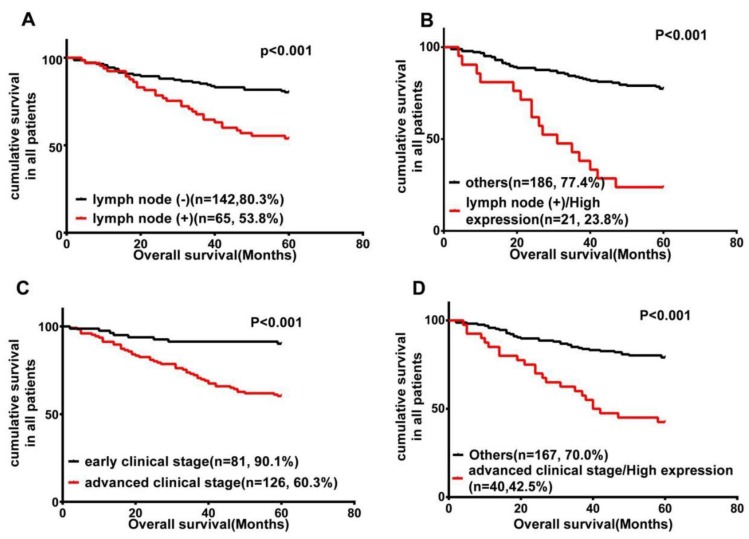
Wnt3a expression is inversely related to LSCC patients' survival. Kaplan-Meier survival analysis of OS in all patients, according to lymph node metastasis status **(A)**; the combination of lymph node metastasis status and Wnt3a expression **(B)**; clinical stage **(C)**; and the combination of advanced clinical stage and Wnt3a expression **(D)**.

It has been widely accepted that lymph node metastasis is significantly associated with a shorter overall survival 3, 4. Our data also helped determine the prognostic value of lymph node metastasis, which was significantly associated with shorter overall survival (P < 0.001; Fig. 3A). Therefore, we further carried out a subset of survival analyses that combined the expression of Wnt3a protein and lymph node metastasis status. These results revealed that patients with phenotype of high Wnt3a expression and positive lymph node metastasis had worse OS than those with other characteristics (P < 0.001; Fig. 3B).

In addition, clinical stage as an independent prognostic risk factor is also an important element influencing shorter OS in this study (P < 0.001; Fig. 3C). We further analyzed the combinational impact of the clinical stage and Wnt3a expression on prognosis. These results disclosed that high Wnt3a expression and advanced clinical stage better predicted a worse overall survival in LSCC patients than either parameter on their own (P < 0.001; Fig. 3D).

## Discussion

In this study, we aimed to explore the role of Wnt3a in the pathogenesis of LSCC. Our results demonstrated that Wnt3a expression was higher in LSCC tissues than that in normal tissues, and that it was also significantly associated with histological grade, clinical stages, and lymph node metastasis. Finally, we found that high Wnt3a expression was an independent adverse prognostic factor for overall survival, especially when combined with advanced clinical stage and lymph node metastasis status. All these data suggest that Wnt3a expression may play an oncogenic role in LSCC.

As previously reported, the aberrant expression of Wnt3a was also found in several human malignancies, including glioblastoma 16, 17, mammary adenocarcinoma 18, 19, colon carcinoma 20, lung cancer 21, prostate cancer 22, malignant mesothelioma 23, esophageal squamous cell carcinoma 24, hepatocellular carcinoma 25-27, and melanoma 28. In these malignant tumors, Wnt3a expression is closely related to tumor growth, metastasis, chemo- / radio- resistance and maintenance of CSC characteristics. These findings suggested that Wnt3a expression is a valuable prognostic biomarker in human malignancy.

Wnt3a is a classical ligand in the canonical Wnt/β-catenin signaling pathway and induces the accumulation of β-catenin. Therefore, it is expected that the theoretically high expression of β-catenin should lead to a poor prognosis in head and neck squamous cell carcinoma. Paradoxically, a negative relationship has been reported between β-catenin cell membrane staining and tumor dedifferentiation, invasion, and metastasis in head and neck squamous cell carcinoma (oropharyngeal cancer) 29. One plausible explanation for these contradictory results is the difference in tumor types. Laryngeal cancer is a special type of head and neck squamous cell carcinoma, and it has a relatively better clinical prognosis compared to other types of head and neck squamous cell carcinoma, such as oropharyngeal cancer 1, 2. These controversial results also reflect the complexity of the regulatory function of Wnt3a protein in the canonical Wnt/β-catenin signaling pathway. On the one hand, the canonical Wnt/β-catenin signaling pathway is also regulated by ligands other than Wnt3a 6. On the other hand, Wnt3a can activate other potential signaling pathways, apart from the classical Wnt/β pathway 30-33. For instance, Wnt3a promotes autophagy in neurons by modulating the GSK-3β-AMPK axis rather than the Wnt/β-catenin pathway 33.

In our investigation, our data showed that higher Wnt3a expression meant worse histological grade, advanced clinical stage, and higher cervical lymph node metastatic potential in LSCC patients. In fact, metastasis was deemed as an essential factor to estimate clinical stages, and it was also prone to occur in less-differentiated malignancies. This result is consistent with those of previous studies investigating colorectal cancer 20, 34, hepatocellular carcinoma^25^, ^26^, lung cancer 21, and melanoma 28, in which overexpression of Wnt3a could significantly enhance the invasion and migration potential of tumor cells by inducing epithelial-to-mesenchymal transition (EMT) and the metastasis-related protein matrix metalloproteinases (MMP)-2, -7, and -9. In addition, Wnt3a has been reported to be involved in the induction of CSC characteristics both *in vitro* and *in vivo* 16, 35. All these evidence clearly reveals that Wnt3a is a potential cancer metastasis-associated protein.

Serum Wnt3a has been previously reported as a useful diagnostic biomarker for hepatocellular carcinoma, particularly in the alpha-fetoprotein (AFP)-negative cases 36. Whether the expression of serum Wnt3a protein in patients with LSCC has an important diagnostic value deserves further investigation. However, that objective is beyond the scope of this study. Previous studies have found that Wnt3a can mediate Wnt signaling pathway activation by both autocrine and paracrine signaling loops in the tumor microenvironment (TME). This causes immunosuppression by preventing the capacity of naïve T-cells to differentiate into effector T-cells. Wnt3a also promotes CSC characteristics in the tumor through the perpetuation of a paracrine signaling loop in cancer cells and cancer-associated fibroblasts (CAF) 10, 35. However, the role of Wnt3a in the tumor microenvironment is not fully understood. We will further explore the effects of Wnt3a on T-cells and macrophages in LSCC through *in vivo* and *in vitro* experiments.

In conclusion, this is the first study to explore the prognostic value of Wnt3a protein in LSCC and found that the upregulated expression of Wnt3a protein is a predictor of multiple cancer malignant processes such as histological grade, advanced clinical stage, and lymph node metastasis. Univariate and multivariate analyses indicate that Wnt3a is an independent prognostic factor in patients with LSCC and is a useful biomarker for assessing lymph node metastasis and prognosis in patients with LSCC. We will further explore the molecular regulatory mechanisms related to Wnt3a, which may lead to Wnt3a becoming a new target of molecular-targeted therapies for LSCC.

## Figures and Tables

**Table 1:** Correlation between Wnt3a expression and clinicopathological parameters

Parameter	Number	Low	Middle	High	χ^2^ value	*P*-value ^a^
Age					0.587	0.746
<58	106	35	48	23		
≥58	116	40	47	29		
Gender					4.717	0.095
Male	213	74	88	51		
Female	9	1	7	1		
Primary tumor site					0.508	0.776
Glottic	148	52	61	35		
Other	74	23	34	17		
T classification					2.066	0.356
T1 + T2	107	40	46	21		
T3 + T4	115	35	49	31		
Histological grade ^b^					10.656	**0.031**
G1	154	56	64	34		
G2	51	17	25	9		
G3	17	2	6	9		
Clinical stage					11.076	**0.004**
I-II	86	39	35	12		
III-IV	136	36	60	40		
Lymph node status					6.993	**0.03**
N0	153	60	62	31		
N+	69	15	33	21		

**(a)** P ≤ 0.05 was considered to be statistically significant and significant P-values were indicated in bold. **(b)** G1, G2, and G3 were defined as well, moderately, and poorly differentiated tumors, respectively.

**Table 2:** Cox model analysis of overall survival

Parameter	Relative risk (95% CI)^a^	P-value^b^
**Univariate**		
Gender	2.785 (0.386-20.112)	0.310
Age	0.795(0.795-2.263)	0.271
Histological grade	0.896(0.581-1.384)	0.622
Primary tumor site	0.470(0.281-0.788)	**0.004**
T classification	2.282 (1.308-3.982)	**0.004**
Clinical stage	4.711(2.232-9.944)	**<0.001**
Lymph node metastasis	2.678 (1.598-4.487)	**<0.001**
Wnt3a expression	1.675 (1.192-2.353)	**0.003**
**Multivariate**		
Clinical stage	3.437 (1.078-10.959)	**0.037**
Wnt3a expression	1.512 (1.057-2.163)	**0.024**

**(a)** Abbreviation: 95% CI, 95% confidence interval. **(b)** P ≤ 0.05 was considered to be statistically significant and significant P-values were indicated in bold.

## Abbreviations

LSCC: laryngeal squamous cell carcinoma
OS: overall survival
TNM: Pathological tumor-node-metastasis
LRP: low-density lipoprotein receptor-related protein
EMT: epithelial-to-mesenchymal transition
MMP: matrix metalloproteinase
TME: tumor microenvironment
CSC: cancer stem cell
CAF: cancer associated fibroblast
